# Polarisation of Tumor-Associated Macrophages toward M2 Phenotype Correlates with Poor Response to Chemoradiation and Reduced Survival in Patients with Locally Advanced Cervical Cancer

**DOI:** 10.1371/journal.pone.0136654

**Published:** 2015-09-03

**Authors:** Marco Petrillo, Gian Franco Zannoni, Enrica Martinelli, Luigi Pedone Anchora, Gabriella Ferrandina, Giovanna Tropeano, Anna Fagotti, Giovanni Scambia

**Affiliations:** 1 Department of Obstetrics and Gynecology, Gynecologic Oncology Unit, Catholic University of the Sacred Heart, Rome, Italy; 2 Department of Oncology, Gynecologic Oncology Unit, Foundation “John Paul II” Catholic University of the Sacred Heart, Campobasso, Italy; 3 Department of Human Pathology, Division of Gynecologic Pathology, Catholic University of the Sacred Heart, Rome, Italy; 4 Division of Gynecologic Oncology, St. Maria Hospital, University of Perugia, Terni, Italy; Ospedale Pediatrico Bambino Gesu', ITALY

## Abstract

**Objective:**

we investigate the prognostic role of pre-treatment ratio between Type 1 (M1) and Type 2 (M2) tumor-associated macrophages (TAMs) in locally advanced cervical cancer (LACC) patients treated with chemoradiation (CT/RT).

**Methods:**

84 consecutive LACC patients treated with cisplatin-based CT/RT for a total dose of 50.0 Gy, followed by radical surgery were analysed. Double-staining immunohistochemistry of CD163/p-STAT, CD68/pSTAT1, CD163/c-MAF, and CD68/c-MAF was performed on tumor samples taken at the time of diagnosis. TAMs with CD163+pSTAT1+, or CD68+pSTAT1+ were defined M1; CD163+c-MAF+ or CD68+c-MAF+ defined the M2 phenotype. The number of M1 and M2 cells was counted at low magnification by evaluating for each case the same tumour area. The ratio between M1 and M2 (M1/M2) was finally calculated.

**Results:**

At diagnosis, we observed a direct correlation between the number of circulating monocytes and of TAMs (p-value = 0.001). Patients with high M1/M2 experienced more frequently complete pathologic response (no residual tumor) to CT/RT, compared to cases with low M1/M2 (55.0% Vs 29.5%; p-value = 0.029). At multivariate analysis M1/M2 (OR = 2.067; p-value = 0.037) emerged as independent predictor of pathologic response to CT/RT. Women with high M1/M2 showed a longer 5-yrs Disease-free (67.2% Vs. 44.3%; p-value = 0.019), and 5-yrs Overall (69.3% Vs. 46.9%; p-value = 0.037) survival, compared to cases with low M1/M2. The presence of a high M1/M2 ratio was independently associated with an unfavourable survival outcome in multivariate analysis.

**Conclusions:**

polarisation of TAMs toward a M2 phenotype, as reflected by a lower M1/M2 ratio, is an independent predictor of poor response to CT/RT, and shorter survival in LACC.

## Introduction

Despite the relevant achievements in the field of primary and secondary prevention, cervical cancer still represents the second most common malignancy in young women [1]. Furthermore, even in developed countries, the proportion of patients with locally advanced disease remained stable in the last decade [2]. From a therapeutic point of view, exclusive concomitant chemoradiation (CT/RT) has been recognized as the standard treatment for patients with locally advanced cervical cancer (LACC) [3–8]. On the other hand, other investigational strategies employing completion surgery after chemotherapy or CT/RT have been also explored with the aim to improve survival [9–12]. However, regardless of the treatment strategy adopted, approximately 30% of women with LACC relapse and die of disease.

For these reasons, given the high variability in term of survival outcome and response to CT/RT, there is a growing demand of reliable prognostic markers able to help clinicians in developing more tailored therapeutic approaches.

In this context, an increased pre-treatment circulating monocyte count has been recently recognized as a predictor of poor prognosis, in patients with squamous cell LACC [13]. These findings strongly suggest the relevant role in cervical cancer progression of the recruitment and differentiation of circulating monocytes into tumor associated macrophages (TAMs). Furthermore, it is well known that TAMs are a very complex population consisting of two phenotypes, each one with specific functions in the tumor microenvironment [14]. When activated by interferon-γ (IFN-γ) or tumor necrosis factor α (TNFα), Type 1 (M1) TAMs promote the inflammatory immune responses by increasing antigen presentation capacity and inducing the Th1 immunity through the production of cytokines such as IL12 [15]. On the other the production by Type 2 (M2) TAMs of TGFβ, PGE2 and the immunosuppressive cytokines IL-10 contributes to a general suppression of antitumoural activities [16,17]. Despite these interesting biological evidences, the clinical role of type 1 (M1), and type 2 (M2) TAMs in women with LACC remains to be established. For these reasons, here we investigate the prognostic role of M1 and M2 TAMs in a large series of LACC patients submitted to CT/RT followed by radical surgery.

## Patients and Methods

### Patients and Treatment

Between March 2009 and December 2011, 84 women with LACC have been admitted (at) to the Gynecologic Oncology Units of Catholic University of Rome. The clinico-pathological characteristics of the study population have been summarized in Table 1. All patients included in the study showed: biopsy-proven carcinoma of the cervix (stage IIB–IVA), no evidence of disease outside the pelvis, Eastern Cooperative Oncology Group performance status < 2, adequate renal and liver function, no prior cancer other than basal cell carcinoma. All patients signed a written informed consent agreeing to be submitted to all the described procedures, and for their clinical data to be prospectively collected and analysed for scientific purpose. The study received the approval of the Ethical Committee of the Catholic University of the Sacred Heart. Pre-treatment work up included collection of medical history, clinical examination, chest radiography, abdominopelvic magnetic resonance imaging (MRI), complete blood count and measurement of liver and renal function, cystoscopy and proctoscopy in case of clinical suspicion of bladder and/or rectum invasion. The results of the monocyte blood count performed at the time of pre-treatment work up were retrieved and analysed in the present study. Neoadjuvant CT/RT was administered as follows: whole pelvic irradiation in 25 fractions (2.0 Gy/day, on weeks 1–2, 5–6, 9, totalling 50 Gy) in combination with cisplatin (20 mg/m^2^, 2-h intravenous infusion) and 5-fluorouracil (1,000 mg/m^2^, 24-h continuous intravenous infusion), both on days 1–4, every 4 weeks, for 3 cycles [18]. All cases were submitted seven/eight weeks after the end of concomitant CT/RT to radical hysterectomy and pelvic±aortic lymphadenectomy. After surgery, patients were submitted to routine follow up procedures. Pathologic response to CT/RT was defined: complete in absence of any residual tumor after treatment (pR0), microscopic (pR1) in presence of only microscopic tumor foci (≤3 mm maximum dimension), and macroscopic (pR2) when we observed persistence of residual tumor >3 mm in maximum dimension [19].

**Table 1:** Distribution of clinico-pathological characteristics at diagnosis and after neoadjuvant chemoradiation in the overall study population, and according with the levels of M1/M2.

Characteristics	All cases	High M1/M2	Low M1/M2	p-value^a^
	Nr. (%)	Nr. (%)	Nr. (%)	
**All cases**	84	40 (47.6)	44 (52.4)	-
**Median Age (years)**	55 (22–79)	52 (25–78)	56 (22–79)	0.132
**Histotype**				
Squamous	71 (84.5)	33 (82.5)	38 (86.4)	
Adenocarcinoma	13 (15.5)	7 (17.5)	6 (13.6)	0.765
**Tumor grade**				
G1/2	48 (57.1)	26 (65.0)	32 (72.7)	
G3	26 (42.9)	14 (35.0)	12 (27.3)	0.485
**FIGO Stage**				
IB2-IIB	68 (82.1)	33 (82.5)	35 (79.5)	
III-IVA	16 (17.9)	7 (17.5)	9 (20.5)	0.786
**Cervical residual tumor after CT/RT** ^**b**^				
pR0	35 (41.6)	22 (55.0)	13 (29.5)	
pR1-pR2	49 (58.4)	18 (45.0)	31 (70.5)	**0.026**
**Histologic lymph nodal status after CT/RT** ^**b**^				
Negative	70 (83.3)	35 (87.5)	35 (79.5)	
Positive	14 (16.7)	5 (12.5)	9 (20.5)	0.389
**Number of TAMs at diagnosis** Median (range)	12 (0–46)	11 (4–41)	14 (0–46)	0.782
**Number of M1 at diagnosis** Median (range)	5 (0–24)	8 (2–24)	3 (0–13)	**0.0001**
**Number of M2 at diagnosis** Median (range)	5 (0–34)	3 (0–20)	10 (0–34)	**0.0001**
**Baseline monocyte count, μl** Median (range)	490 (230–1180)	500 (24–1180)	510 (23–820)	0.427

^a^Calculated by Mann-Whitney non parametric test

^b^CT/RT = chemoradiation

### Immunohistochemical Assessment of TAMs

As recently published, we used the expression of pSTAT1, and c-MAF in the context of CD68 or CD163 to characterise the *in situ* polarisation of TAMs [20]. In particular, M1 phenotype was defined as the concomitant expression of pSTAT and CD68, or pSTAT and CD163; while TAMs with M2 phenotype were identified using the combination of c-MAF and CD68, or c-MAF and CD163 [20].

Double staining immunohistochemistry of CD163/pSTAT1, CD68/pSTAT, CD163/c-MAF, CD68/c-MAF were performed on Formalin-Fixed, Paraffin-Embedded (FFPE) cervical cancer samples taken at the time of diagnosis, before the start of CT/RT. Three μm paraffin tissue sections mounted on poly-l-lysine- coated slides and dried at 37°C overnight were used. Sections were taken form FFPE areas showing an homogenous and large distribution of cancer tissue covering almost the entire slide. The slides were deparaffinised in xilene and re-hydrated conventionally, the endogenous peroxidase was blocked with 3% H_2_O_2_ in H_2_O for 5 min. Antigen retrieval procedure was performed by microwave oven heating in EDTA pH8 (2 times for 4 min.). To reduce non-specific binding the sections were incubated with 20% normal goat serum for 30 min at room temperature. Cells expressing CD163 or CD68 were identified after 1h incubation at room temperature by using the monoclonal antihuman CD163 antibody (1:50; clone10D6; BIOCARE), and the monoclonal antihuman CD68 antibody (1:50; clone PG-M1; DAKO), respectively. Detection was performed using a labelled polymer En Vision-mouse+ System-HRP (DAKO, Carpinteria, CA, USA), 30 min at room temperature. Diaminobenzidine was used as a chromogen (DAB substrate System, DAKO). Subsequently, slides were washed (TBS1X Ph7,5) for 5 minutes and the appropriately diluted c-MAF (polyclonal antihuman c-MAF; 1:50; Santa Cruz) or pSTAT1 (polyclonal antihuman antibodies pSTAT; 1:50; Santa Cruz) were incubated overnight at 4°C. Detection was achieved using a labelled polymer En Vision-rabbit+ System-AP (DAKO, Carpinteria, CA, USA), 30 min at room temperature. Fast Red was used as a chromogen (UCS diagnostic srl). Sections were counterstained with haematoxylin and mounted with Glycergel (DAKO). The number of labelled M1 and M2 macrophages was counted manually by two different researchers (GFZ; EM) without any prior knowledge of the clinical and biological parameters, at low magnification (5X objective lens) by evaluating for each case the same tumour area consisting of ten representative high-power fields (400x magnification) of tissue sections. Finally the M1/M2 ratio was calculated.

### Statistical Analysis

Linear regression model was used to investigate the role of clinicopathological variables as predictors of residual disease after CT/RT. Overall survival (OS) and disease-free survival (DFS) were calculated from the date of diagnosis to the date of death/recurrence or date of last follow-up visit, according to the M1, M2 and M1/M2 levels. Medians and life tables according to TAMs levels were computed using the product-limit estimate by the Kaplan and Meier method, and the log-rank test was employed to assess the statistical significance [21,22]. All statistical calculations were performed using the Statistical Package for Social Sciences (Version 17.0, SPSS Inc., Chicago, IL, USA).

## Results

**Fig 1:**
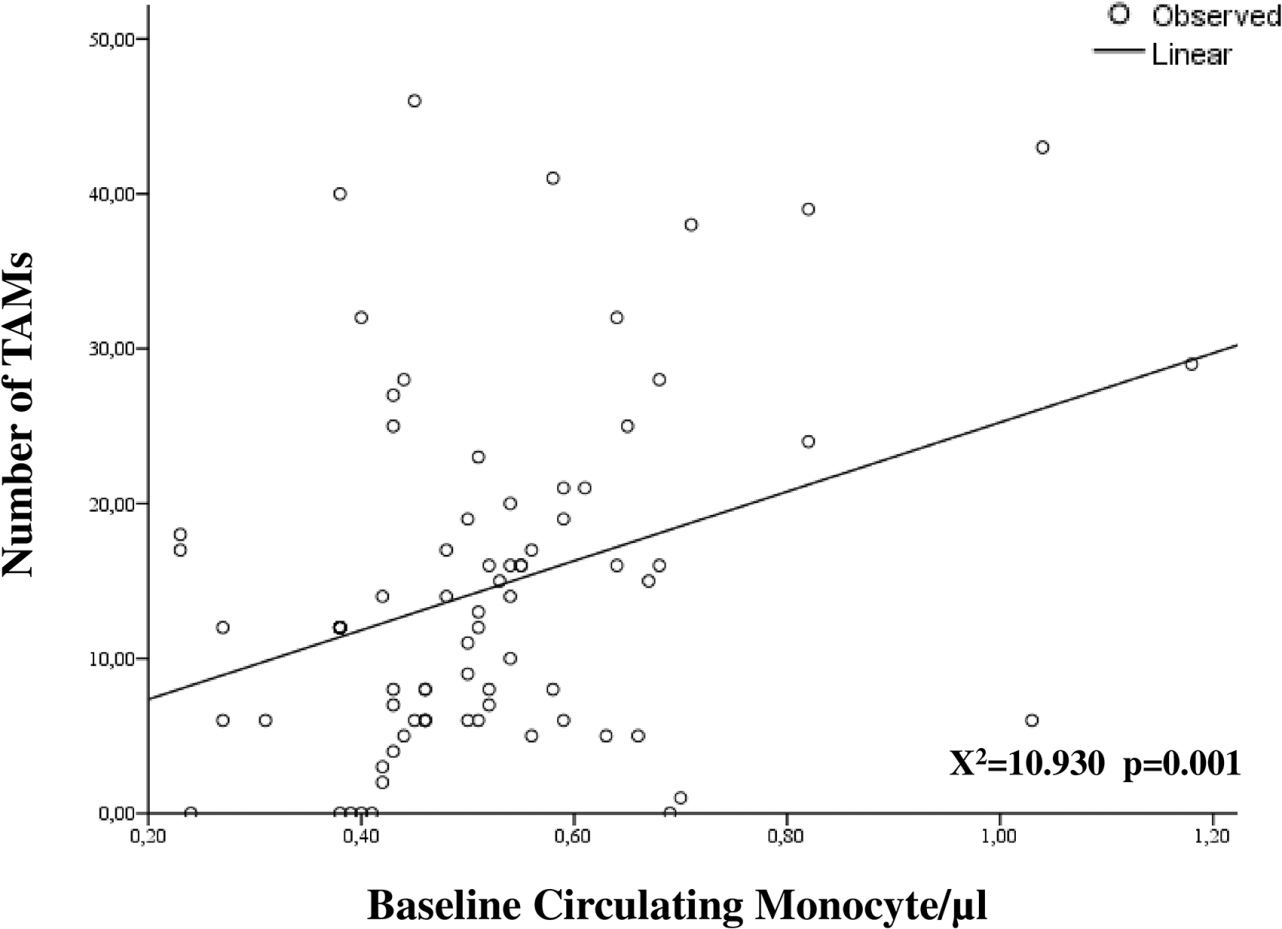
Correlation between the number of TAMs and circulating monocyte count at diagnosis. Empty circles represent the number of TAMs and circulating monocyte in each patient, linear trend has been also provided (*p value*, and *X* ^*2*^ have been calculated applying linear regression model).

The clinico-pathological characteristics of the whole series have been summarized in Table 1. Sixty-eight patients (82.1%) showed FIGO stage IB2/IIB, while 16 cases (17.9%) had stage III-IVA. The vast majority of women (84.5%) showed a squamous histotype, and complete pathologic response to CT/RT was documented in 35 patients (41.6%). The median number of TAMs detected at immunohistochemical analysis in tumor biopsies taken at the time of diagnosis was 12 (0–46), and the median number of M1 and M2 macrophages was equal accounting for 5 (Table 1). The ratio between the number of M1 and M2 was calculated for each patient, and median value was 0.600, ranging from 0 to 19. Median monocyte count at baseline evaluation was 490/μl (230–1180), and number of circulating monocytes showed a direct statistically significant correlation with number of TAMs (p-value = 0.001; Fig 1).

**Fig 2:**
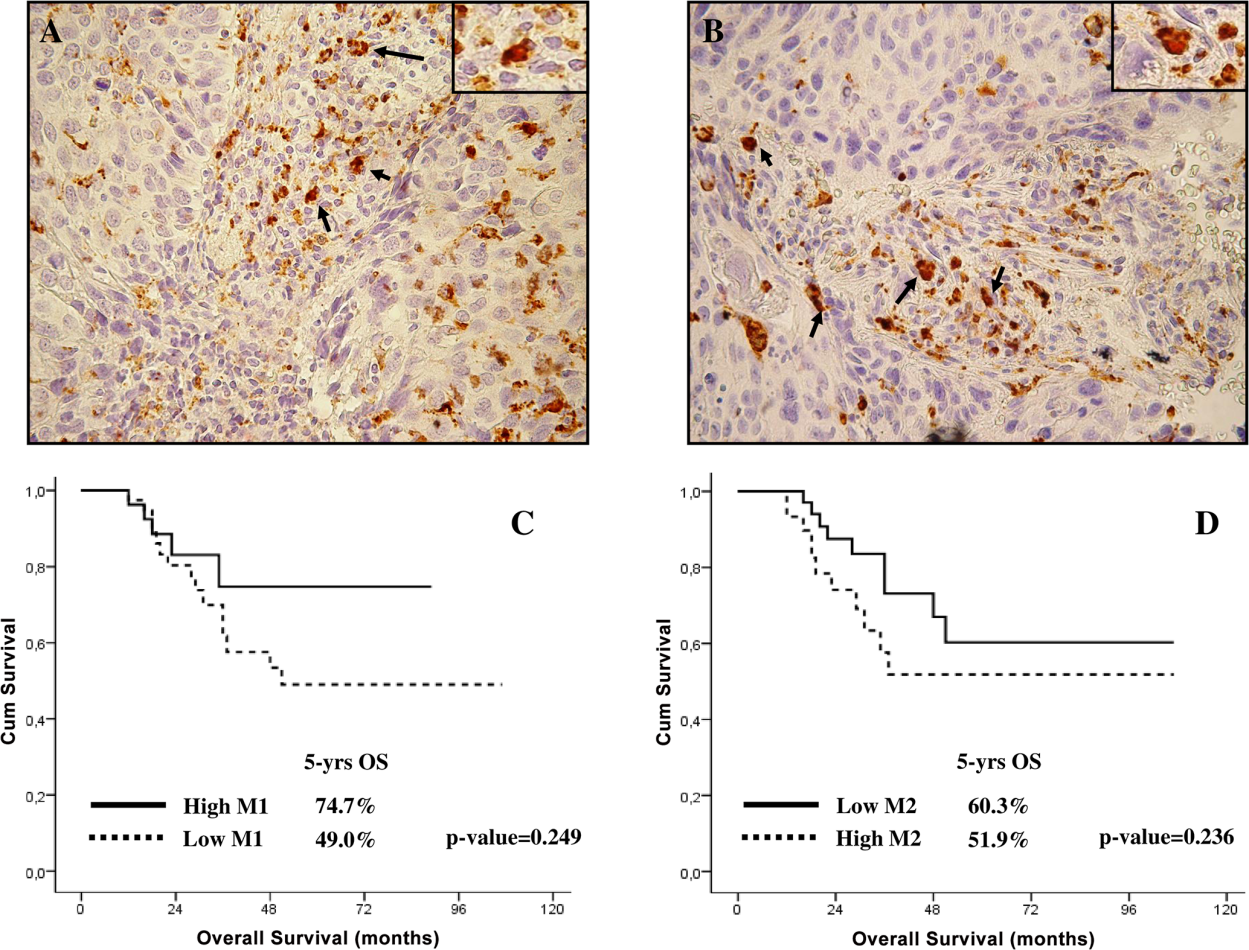
A-B)Examples of double staining immunohistochemistry to identify M1 and M2 TAMs in cervical cancer patients. Expression of CD68 is indicated by brown cytoplasmic/membranous staining. The expression of transcription factors pSTAT and c-MAF is indicated by red nuclear staining. Representative examples have been presented of cervical cancer patients with high numbers of M1 (CD68+pSTAT1+) cells (A; arrows indicate M1 cells), and M2 (CD68+c-MAF+) cells (B; arrows indicate M2 cells) (original magnification 200X; insert magnification 1000X). C) Overall survival curves in LACC patients with high and low M1 levels (solid line = high M1, dashed line = low M1). D) Overall survival curves in LACC patients with high and low M2 levels (solid line = high M2, dashed line = low M2).

The median values of M1, M2, and M1/M2 were used as thresholds to identify patients with low and high levels. Representative examples of cases with high M1 and M2 levels have been provided in Fig 2A and 2B.

As shown in Table 1, no differences were documented in term of clinico-pathological characteristics between women with high a low M1/M2. However, the percentage of women showing pR0 after CT/RT was almost double in the group of patients with high M1/M2 compared to cases with low levels of the ratio (55.0% Vs 29.5%; p-value = 0.029). At univariate/multivariate analysis, squamous histotype (OR = 2.120; p-value = 0.028), and high levels of the M1/M2 (OR = 2.067; p-value = 0.037) ratio emerged as the only independent predictors of pR0 (Table 2).

**Table 2:** Univariate and multivariate analysis of clinical-pathological parameters as predictors of complete pathologic response after preoperative chemoradiation.

	Univariate analysis		Multivariate analysis	
Variable	OR	p-value^a^	OR	p-value
**Age (years)** ^**b**^	0.867	0.974	-	-
**Histotype**				
Squamous				
Adenocarcinoma	2.467	0.025	2.120	**0.028**
**Tumor Grade**				
G1-2				
G3	0.655	0.123	-	-
**FIGO Stage**				
IB2-IIB				
III-IVA	0.923	0.851	-	-
**Baseline monocyte count** ^**b**^	0.877	0.455	-	-
**Number of TAMs**				
Low				
High	0.641	0.165	-	-
**Number of M1**				
Low				
High	1.316	0.192	-	-
**Number of M2**				
Low				
High	0.418	0.161	-	-
**M1/M2**				
Low				
High	2.183	0.019	2.067	**0.037**

^a^Only variables with a p value<0.05 at univariate analysis were included in multivariate analysis.

^b^Included in the model as continuous variable.

### Survival Analysis

As of June 2014, the median duration of follow-up was 28 months (7–107) in the overall series. Recurrence and death from disease were observed in 21 and 20 patients, respectively.

Women with high M1 levels showed a longer 5-yrs DFS (79.7% Vs 51.3%; p-value = 0.450), and 5-yrs OS (74.7% Vs 49.0%, p-value = 0.249; Fig 2C) compared to cases with low M1 levels, but these differences did not reach the statistical significance. On the other, the presence of a high number of M2 was associated with a shorter 5-yrs DFS (54.3% Vs 64.8%; p-value = 0.067), and OS (51.9% Vs 60.3%, p-value = 0.236), but again the differences were not statistically significant (Fig 2D).

**Fig 3:**
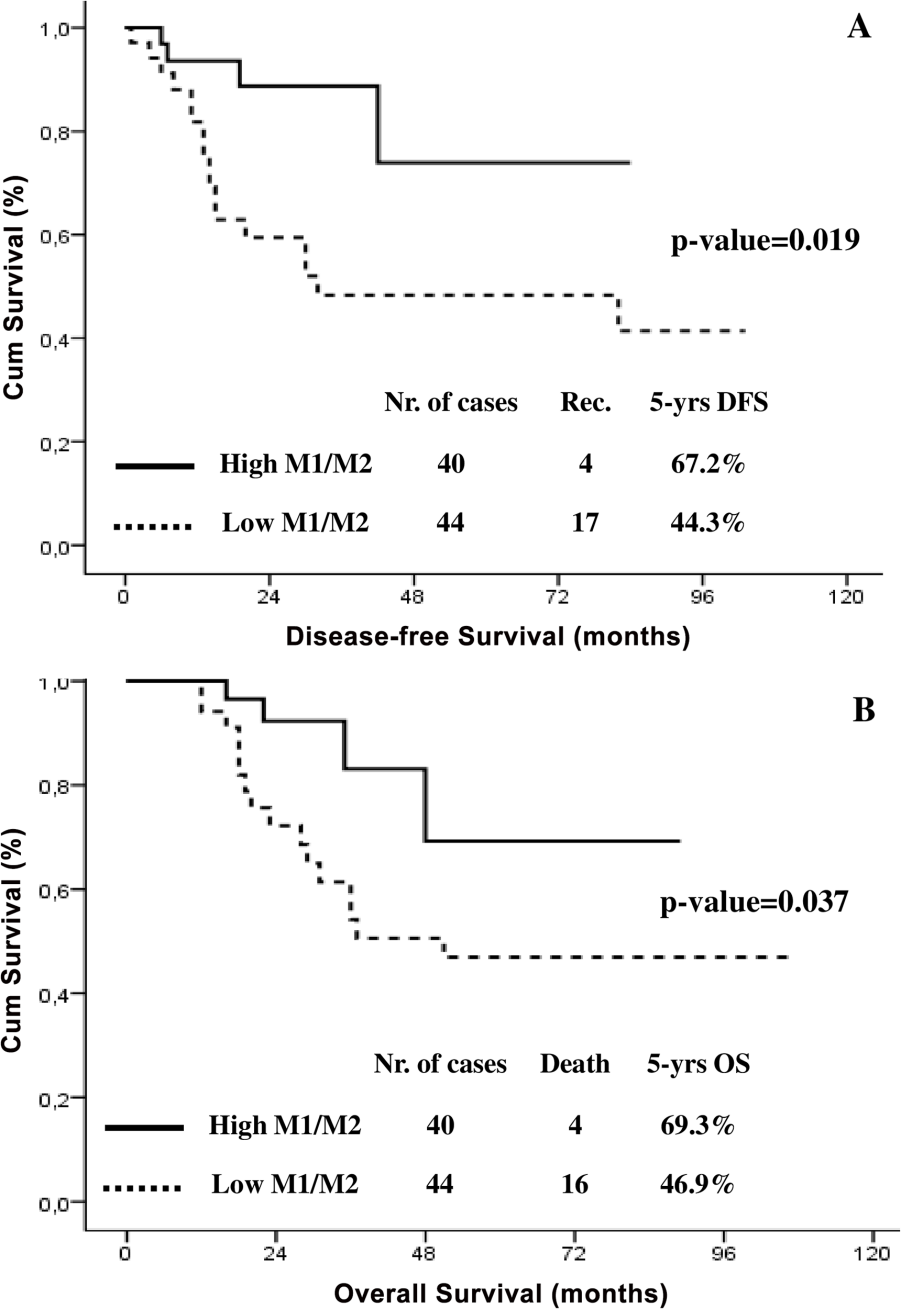
Disease-free (A) and Overall (B) survival curves in LACC patients with high and low M1/M2 levels (solid line = high M1/M2, dashed line = low M1/M2).

Given the opposite impact on survival of M1 and M2 phenotypes, we focused our attention on prognostic role of the M1/M2 ratio. 5-yrs DFS was significantly longer in women with high M1/M2, compared to cases with predominance of M2 (i.e. low M1/M2) (67.2% Vs. 44.3%; p-value = 0.019; Fig 3A). Similarly, the presence of a high M1/M2 was associated with a prolonged 5-yars OS compared to cases with low M1/M2 (69.3% Vs. 46.9%; p-value = 0.037; Fig 3B). With the aim to analyse the independent prognostic role of TAMs polarisation, Cox’s regression model was applied to survival data. Both in univariate and multivariate analysis, levels of M1/M2 ratio (DFS: HR = 0.233, p-value = 0.028; OS: HR = 0.245; p-value = 0.030), as well as baseline monocyte count (DFS: HR = 1.462, p-value = 0.043; OS: HR = 1.181; p-value = 0.041) were independently associated with a reduced DFS, and OS (Table 3).

**Table 3:** Univariate and multivariate analysis of clinical-pathological parameters as predictors of disease-free and overall survival.

	Disease Free Survival		Overall Survival	
	Univariate^a^	Multivariate	Univariate^a^	Multivariate
		p-value (HR)		p-value (HR)
**Age (years)** ^**b**^	0.627	-	0.841	-
**Histotype**				
Squamous				
Adenocarcinoma	0.754	-	0.824	-
**Tumor Grade**				
G1-2				
G3	0.453	-	0.857	-
**FIGO Stage**				
IB2-IIB				
III-IVA	**0.024**	**0.033 (2.810)**	**0.032**	**0.037 (2.982)**
**Cervical residual tumor after CT/RT**				
pR0				
pR1-pR2	**0.006**	**0.021 (2.983)**	**0.002**	**0.015 (3.137)**
**Baseline monocyte count** ^**b**^	**0.033**	**0.043 (1.462)**	**0.023**	**0.041 (1.181)**
**Number of TAMs**				
Low				
High	0.342	-	0.135	-
**Number of M1**				
Low				
High	0.456	-	0.259	-
**Number of M2**				
Low				
High	0.076	-	0.244	-
**M1/M2**				
Low				
High	**0.016**	**0.028 (0.233)**	**0.025**	**0.030 (0.245)**

^a^Only variables with a p value<0.05 at univariate analysis were included in multivariate analysis.

^b^Included in the model as continuous variable.

## Discussion

After the advent of CT/RT relevant advances have been achieved in prognosis of LACC. However, it still exists a cohort of women with reduced survival due to the presence of tumor cells resistant to chemo and radiation therapy.

Recently, results of a retrospective analysis conducted in a large series of women with locally advanced disease demonstrated that high pre-treatment circulating monocyte count is an independent negative prognostic factor and it can be used as adjunctive biomarker to SCC-antigen [13]. In our study, we confirmed these findings (Table 3), demonstrating for the first time the existence of a significant correlation between baseline circulating monocyte count and the number of TAMs in tumor samples taken at the time of diagnosis (Fig 1). These results strongly support the existence of a circulating monocyte/TAMS axis, which acts in regulating cervical cancer progression [23].

It is well known that the tumour microenvironment represents a relevant source of chemokines, which drive the extravasation of circulating monocytes into the tumour site [23]. After reaching the malignant tissue, a complex network of cytokines, including IL-4, IL-10, and IL-13, induces the differentiation of monocytes into M1, and M2 TAMs [23]. In this context, our study represents the first evaluation of the clinical role of M1, and M2 TAMs in patients with LACC.

First, we report in our study a ratio between M1 and M2 of 0.600, thus suggesting that at diagnosis the tumor microenvironment is mainly polarized toward an M2 phenotype. These data are in keeping with compelling evidences suggesting that M2 macrophages are involved in the process of cancer development in several human malignancies, including cervical cancer [14]. Mechanisms by which this occurs are still unclear so further studies to investigate and to clarify them are desirable.

Moreover, we observed that the percentage of women showing pR0 after CT/RT is almost double in patients with high M1/M2 compared to cases with a low M1/M2, and ratio between M1 and M2 macrophages represents an independent predictor of pR0. These data emphasize that differentiation of TAMs toward an M2 phenotype can promote the development of resistance to CT/RT in LACC patients. Our results are strongly supported by recent evidences, which suggest that inflammation promotes the differentiation of M2 macrophages, which ultimately drive resistance to platinum agents in cervical cancer cell lines [24].

Focusing on survival, we did not observe a correlation between the total number of TAMs and survival (Table 3). These results can be easily explained considering that the population of TAMs consists of at least two subtypes of macrophages with opposite functions [14]. In fact, the presence of a high number of M1 was associated with a not significant trend toward a longer survival, while increased M2 levels were associated with a shorter survival, again without reaching statistical significance (Fig 2C and 2D). On the other hand, ratio between M1 and M2 levels clearly emerged as a very interesting relevant independent negative prognostic factor, with an around 20% increase of 5-yrs DFS and OS in women with high level of M1/M2. Recently, the presence of a strong intraepithelial infiltration of M1 macrophages has been associated with a large influx of T lymphocytes and an improved survival in cervical cancer [25]. In keeping with these *in vitro* evidences, our results provide the first clinical demonstration that, (rather) more than the total number of M1 and M2 cells, the polarisation of TAMs toward a specific phenotype determines the tumor biological features and patients' prognosis.

From a clinical point of view, it has to be considered that an increased number of TAMs has been associated with a higher microvessel density [26], and enhanced VEGF production [27] in cervical cancer patients. Therefore, it will be useful to investigate in the future the impact of the polarisation of TAMs on tumor angiogenesis, particularly after the introduction of Bevacizumab in the management of cervical cancer patients [28]. These experimental and clinical results prompted us to further evaluate potential targets that exhibit tumorigenic and immunosuppressive potential related to tumour associated macrophages. Furthermore, our findings support further preclinical evaluations of the efficacy of the drugs targeting TAMs, such as Trabectedin, in cervical cancer [29].

We acknowledge that our study represents one of the first applications in cervical cancer patients of an immunohistochemical approach to determine the *in situ* the number of M1 and M2 TAMs; however, the reliability of our experimental protocol is supported by previously published evidences [20].

Despite the potential relevance of our data several limitations affect our study: the small sample size, the not-standardized approach of our experimental protocol suggest that further prospective investigations are required prior to draw definitive conclusions.

In conclusion, our results demonstrate that the polarisation of TAMs toward an M2 phenotype at diagnosis, as reflected by a lower ratio between M1 and M2 macrophages, is a very interesting marker of poor response to CT/RT and shorter survival in women with LACC. Our findings emphasize the need to carry on further experimental investigations with molecular based approach in order to clarify the role in cervical cancer of the specific subtypes of TAMs, including also the population of mixed M1 and M2 TAMs which has been recently demonstrated as involved in promoting tumor growth in colorectal cancer [30].
